# Validity and reliability of measurement of peripheral oxygen saturation during the 6-Minute Walk Test in patients with systemic sclerosis

**DOI:** 10.1007/s00296-024-05532-5

**Published:** 2024-02-10

**Authors:** Amanda Lynggaard Elkjær, Esben Uggerby Næser, Katja Thorup Aaen, Henrik Lynge Hovgaard, Peter Juhl-Olsen, Elisabeth Bendstrup, Klaus Søndergaard

**Affiliations:** 1 The Department of Rheumatology, Aarhus University Hospital, Palle Juul-Jensens, Boulevard 59, 8200 Aarhus N, Denmark; 2 The Department of Anaesthesiology and Intensive Care, Aarhus University Hospital, Palle Juul-Jensens, Boulevard 99, 8200 Aarhus N, Denmark; 3 The Department of Clinical Medicine, Aarhus University, Palle Juul-Jensens Boulevard 91, 8200 Aarhus N, Denmark; 4 The Department of Respiratory Diseases and Allergy, Aarhus University Hospital, Palle Juul-Jensens, Boulevard 99, 8200 Aarhus N, Denmark

**Keywords:** Reproducibility of Results, Oximetry, Walk Test, Scleroderma, Systemic

## Abstract

Peripheral oxygen saturation (SpO_2_) using the fingers may have important limitations due to Raynaud’s phenomenon and sclerodactyly in patients with systemic sclerosis (SSc). Sensors located at more central body positions may be more accurate as these as less prone to Raynaud attacks. To determine the validity and reliability of the SpO_2_ measured at the finger, forehead, and earlobe during the 6-Minute Walk Test (6MWT). Eighty two patients with SSc had an arterial line placed while performing the 6MWT. Peripheral oxygen saturation was simultaneously measured by finger, forehead, and earlobe sensors and compared to the arterial oxygen saturation (SaO_2_) measured before and after the 6MWT. 40 patients repeated the 6MWT one week later to determine re-test reliability. We used Bland–Altman plots to display the agreement between SpO_2_ and SaO_2_. The intraclass correlation coefficient for repeated measurement of minimum SpO_2_ was calculated. The mean difference between SpO2 and SaO2 after the 6MWT was − 3% (SD: ± 5), 0% (SD: ± 2), and 1% (SD: ± 2) for the finger, forehead, and earlobe, respectively. The minimum SpO_2_ measured at the finger demonstrated the poorest re-test reliability: The ICC (95% CI) showed good agreement using the ear and forehead probe (ICC_ear_ = 0.89 [95% CI 0.80; 0.94]; ICC_forehead_ = 0.77 [95% CI 0.60; 0.87]), while a modest reliability was found using the finger probe (ICC_finger_ = 0.65 95% CI [0.43; 0.80]). SpO_2_ should be measured using either the earlobe or forehead during the 6MWT in patients with SSc. Clinical Trials.Gov (NCT04650659).

**Supplementary Information:**

The online version contains supplementary material available at 10.1007/s00296-024-05532-5.

## Introduction

Systemic sclerosis (SSc) is a progressive chronic connective tissue disease characterized by microvasculopathy and extensive fibrosis in the skin and internal organs. The disease has an increased mortality, with SSc associated interstitial lung disease (SSc-ILD) and pulmonary arterial hypertension (SSc-PAH) accounting for the majority of SSc-related deaths [1]. The peripheral microvasculopathy in SSc leads to poor perfusion of the fingers, which manifest as Raynaud’s phenomenon, digital ulcers, and poor healing. Overt symptoms of fibrosis include thickness of the skin, dry skin and contractures of the joints [2]. Many patients with SSc have decreased exercise tolerance, which may have multiple aetiologies, in which musculoskeletal disease, internal organ involvement, and deconditioning may play a role [3]. The disease progression is highly variable, and accurate markers of disease activity is essential for qualified management of the disease.

The 6-Minute Walk Test (6MWT) is a standardized non-invasive sub-maximum exercise test. During the 6MWT, the distance (6MWD), effort, and peripheral oxygen saturation (SpO_2_) are registered [4]. The test is primarily used as an outcome measure of clinical SSc trials, to monitor treatment response in patients with pulmonary involvement, and as a measure of functional capacity in general [5].

Exercise-induced desaturation during the 6MWT is associated with the degree of dyspnea, diffusion capacity for carbon monoxide and the extent of lung fibrosis HRCT in patients with SSc [6–9]. Furthermore, SpO_2_ desaturations have been associated with progression of SSc-ILD and poorer prognosis in patients with SSc [10, 11].

While digital sensors are commonly used to measure SpO_2_ during the 6MWT, these measurements may have important limitations in patients with SSc due to disease related microangiopathy, Raynaud’s phenomenon, sclerodactyly and motion artifacts during the 6MWT [12]. Consequently, finger probe pulse oximetry may cause inaccurate measures of SpO_2_, and there may be substantial variation of SpO_2_ measurements in patients with SSc [13, 14]. Indeed, several authors author advocate for measuring Sp0_**2**_ at the forehead in patients with SSc [5, 7]. Still, the evidence for measuring Sp0_**2**_ at more central locations is based on only a single study examining the re-test reliability of SpO_2_ measurement during the 6MWT in a small cohort of patients with SSc [7].

We aimed to determine the validity and re-test reliability of peripheral oxygen saturation measured at the finger, forehead, and earlobe as compared with blood gas analysis during the 6MWT in patients with SSc.

## Methods

### Study population and study design

We conducted a cross-sectional study at the Department of Rheumatology at Aarhus University Hospital in Denmark from 27 July 2021 to 21 December 2021 involving adult patients diagnosed with SSc according to the ACR/EULAR 2013 criteria [15].

Patients were excluded in case of recent or ongoing pneumonia, pregnancy, a diagnose of connective tissue overlap syndrome [16] or in case of severe physical or mental comorbidity, which prevented the performance of the 6MWT. Patients were allowed to use supplemental oxygen or walking aid during the 6MWT if needed.

### 6MWT and measurements of oxygen saturation

The 6MWT was performed at room temperature by the same investigator (ALE) according to the American Thoracic Society guidelines [4]. Patients had acclimatised and rested for minimum 20 min before test start. SpO_2_, 6MWD and Borg dyspnoea score were collected [4, 17]. Raynaud’s attacks during the 6MWT were noted in case the patient fingers turned white and/or blue. SpO_2_ was continuously measured during the 6MWT by pulse oximeters (*Vyntus® WALK,* Nonin Model 3150, Viare Medical, Germany) using sensors at the finger, earlobe and forehead [18]. The pulse oximeters measured SpO_2_ as integers. The accuracy of the SpO_2_ measurement (interval of 70–100%) with low perfusion was ± 2%. At the first visit, an arterial line was placed in all patients by a trained anesthesiologist (HH or PJ) on the opposite arm of the finger oximetry sensor. Arterial blood was drawn immediately before (pre-exercise) and after (post-exercise) the 6MWT and analysed with a blood gas analyser (ABL800, Radiometer Medical, Brønshøj, Denmark).

A subgroup of patients (*n* = 40) repeated the 6MWT one week later without an arterial line. At visit 2 the earlobe and finger probe were placed on the same earlobe and finger as in visit 1 (Supplementary Fig. 1).

### Data quality

The oximeters were electronic and paired with tablets via Bluetooth, which generated a graph of the continuous measurement of SpO_2_ during the 6MWT (Supplementary Fig. 2). The quality of data was assessed by ALE using the following pre-specified criteria for exclusion of data (Supplementary Table 1).

(1) Technical error in collection or transfer of data from pulse oximeter and tablet (i.e., no readings from pulse oximeter or no data transfer from pulse oximeter to tablet at time of arterial blood gas test).

(2) Technical error in performance or analyse of arterial blood gas test.

In case of doubt, a consensus was reached in cooperation with KS.

### Clinical and paraclinical parameters

The following clinical and paraclinical parameters were collected from the electronical patient record (MidtEPJ, Systematics, Aarhus, Denmark): SSc-disease characteristics, (ii) medication, (iii) modified Rodnan Skin score (mRSS), (iv) routine blood samples, (v) electrocardiogram (ECG), (vi) the latest pulmonary function test (PFT, median time since latest PFT was 10 months [interquartile interval(IQI): 2–18]), (vii) the latest high-resolution computed tomography (HRCT, median time since latest HRCT was 72 months [IQI: 20–113]), (viii) the latest transthoracic echocardiography, (ix) PAH detected by right-heart catheterisation, and (x) comorbidities. SSc-ILD was defined according to the HRCT criteria for ILD patterns [19].

All patients had Nailfold Videocapillaroscopy (NVC) images recorded of the 2nd to 5th finger. The capillary density was assessed with the 90-degree method from minimum 3 images per patient. The general capillary density was defined as the mean capillary density of the available pictures from the same hand.

### Patient-related outcome measures

Patients answered two self-reporting questionaries: Raynaud’s attacks the last month, including the Raynaud’s Condition Score (RCS) [20] and burden of ischaemic ulcers, and the Scleroderma Health Assessment Questionnaire (SHAQ) [21].

### Statistical analysis

Categorial data are reported as counts and percentages, and continuous data as mean values and standard deviation (± SD) when normally distributed or otherwise as median values and interquartile interval (IQI). Data distribution was investigated Q–Q plots and histograms.

The agreement of the SpO_2_ of the three anatomical sites was examined using Bland–Altman plots to display the difference between SpO_2_ and SaO_2_ (bias) at pre-exercise and at post-exercise [22], and the re-test reliability was examined using Bland–Altman plots to display the agreements between the minimum SpO_2_ during the 6MWT, the 6MWD and Borg dyspnea score at visit 1 and visit 2. Furthermore, the overall accuracy of the peripheral oxygen measurement was calculated by the accuracy root mean square (A_rms_) (√ [(bias)^2^ + (precision)^2)^ [23]. In accordance with the Food and Drug Administration recommendation, we used a cut-off of A_rms_ < 3% as the acceptable accuracy of the SpO_2_ measurements [24].

The intraclass correlation coefficient (ICC, 95% confidence interval [95% CI]) for repeated measurement of minimum SpO_2_ was calculated based on an absolute agreement, two-way mixed effect model [25]. The ICC values were interpreted using the following definitions: ICC < 0.5: poor reliability, ICC: 0.5–0.75: moderate reliability, ICC 0.75–0.9: good reliability, and ICC > 0.90: excellent reliability. Furthermore, we calculated the frequency of measurement error of SpO_2_ at post-exercise for the finger, forehead, and earlobe sensors. Measurement errors was defined as values being ± 4% different from SaO_2_ values. In explorative analysis, we examined the impact of demographic parameters, comorbidities, and SSc specific parameters on the risk of measurement errors of the SpO_2_. Statistical significance was tested using Student’s *t* test, the nonparametric Mann–Whitney *U* test and Fisher’s exact test. All analyses were carried out in Stata17, where *p* < 0.05 was considered statistically significant.

### Ethical permissions

The research project was approved by the Central Denmark Region Committees on Health Research Ethics (1-10-72-203-20) at the 30 October 2020 and listed in the Central Denmark Region register of internal research projects (1-16-02-270-20) at the 15 June 2020. ClinicalTrials.gov identifier: NCT04650659.

## RESULTS

### Patient characteristics and baseline characteristics

One hundred ninety-nine patients were screened for the study. Sixteen patients were excluded from the study, and 101 patients chose not to participate. In total, 82 patients participated in our study (Supplementary Fig. 3).

The patients had a median age of 58 years (IQI: 52–66), and 76% of patients were female (Table 1). Thirty-seven patients (45%) had either SSc-ILD or PAH. None of the included patients needed supplemental oxygen or walking aid during 6MWT.

**Table 1:** Baseline characteristics of patients (*n* = 82)

Variable		
**Demographics**		
Age, years, median (IQI)	58	(52–66)
Females, *n* (%)	62	(76%)
Smoking, *n* (%)		
Current smokers	30	(37%)
Ex-smokers	44	(54%)
Never smokers	8	(10%)
**Systemic sclerosis disease characteristics**		
Disease duration*, years, median (IQI)	8	(4–12)
Skin involvement, *n* (%)		
Limited cutaneous	42	(51%)
Diffuse cutaneous	38	(46%)
Sine scleroderma	2	(2%)
mRSS**, median (IQI)	4	(2–6)
mRSS** on fingers only, median (IQI)	1	(1–2)
Autoantibodies, *n* (%)		
ACA	34	(42%)
Anti-SCL-70	15	(18%)
Anti-RNA polymerase III	3	(4%)
Anti U1 RNP	8	(10%)
Anti Th/To	2	(2%)
Organ manifestations, *n* (%)		
Gastrointestinal	69	(84%)
Arthritis	27	(33%)
Myositis	13	(16%)
ILD	35	(43%)
Pulmonary hypertension	2	(2%)
SHAQ: Disease severity, median (IQI)	1	(0–2)
**Results of pulmonary tests**		
Latest lung function parameters, median (IQI)		
FEV_1_, % predicted	96	(85–107)
FVC, % predicted	104	(93–115)
DLCO, % predicted	69	(52–84)
**Latest high-resolution CT-scan,** ***n*** **(%)**		
Sign of interstitial lunge disease	35	(42.7%)
Extent of interstitial features [36] (*n* = 35)		
0–15 %	16	(46%)
15–25 %	6	(17%)
> 25 %	12	(34%)
Unknown	1	(3%)
**Echocardiography,** ***n*** **(%)**		
Reduced left ventricular ejection fraction	2	(2%)
Diastolic dysfunction	8	(10%)
**Respiratory symptoms,** ***n*** **(%)**		
NYHA I	41	(6%)
NYHA II	25	(33%)
NYHA III / IV	15	(18%)
Unknown	1	(1%)
**Microvascular disease of the fingers**		
RCS, median (IQI)	3	(0–6)
History of ischemic digital ulcers, *n* (%)	28	(31%)
Current use of vasodilator, *n* (%)	64	(78%)
**Comorbidity,** ***n*** **(%)**		
Cardiovascular disease	28	(34%)
COPD and/or asthma	9	(11%)
Arthrosis in the knees and/or hips	15	(18%)
Diabetes mellitus	3	(4%)

*Disease duration among 60/82 who could recall years of first SSc-related symptom (not Raynaud Syndrome)

**mRSS evaluated at the latest clinical visit

*IQI* Interquartile interval, *mRSS* modified Rodnan Skin Score, *ACA* Anti-Centromere Antibody, *Anti-SCL-70* Anti-Topoisomerase I, *SHAQ* Scleroderma Health Assessment Questionnaire, *FEV1* Forced Expiratory Volume in 1 s, *FVC* Forced Vital Capacity, *DLCO* Diffusing Capacity of Carbon Monoxide, *ILD* Interstitial lung disease, *NYHA* New York Heart Association, *RCS* Raynaud’s Condition Score, *COPD* Chronic obstructive pulmonary disease and *SSc* Systemic sclerosis

The median 6MWD was 564 m (IQI: 502–622) during the first 6MWT, while the SaO_2_ was 97% (IQI: 97–98) and 97% (IQI: 96–98) at pre- and post-exercise (Supplementary Table 2), respectively. During the 6MWT, 46 of the patients (56%) had visual signs of Raynaud’s attack, and desaturation below 88% were registered in 28%, 17% and 8% of the patients the by probes on the finger, forehead, and earlobe respectively (Supplementary Table 2).

### Validity of peripheral oxygen measurement

**Fig. 1:**
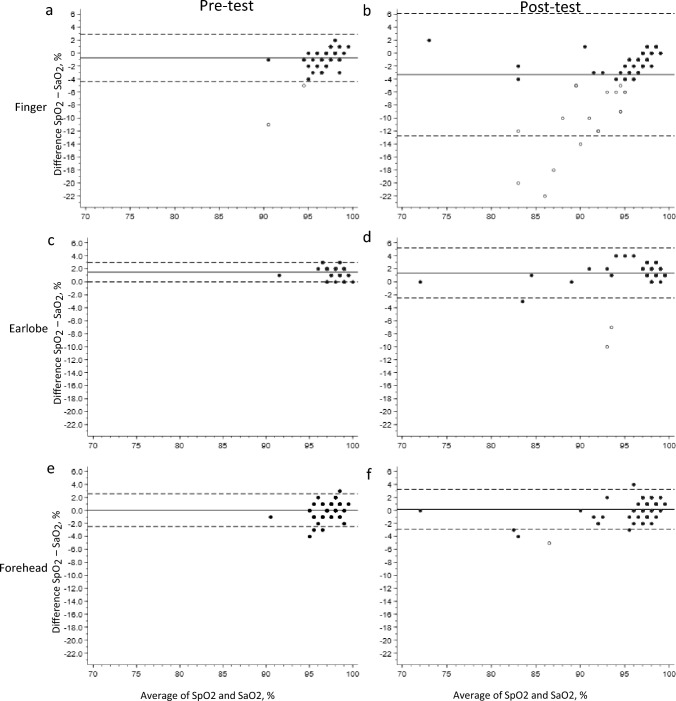
Bland–Altman plots showing the validity of measurement of peripheral oxygen saturation at the finger, earlobe, and forehead. Black circles: values with ≤  ± 4% difference between SpO_2_ and SaO_2_ values. Hollow circle: values with >  ± 4% difference between SpO_2_ and SaO_2_ values. Solid line: Mean difference between SpO_2_ and SaO_2_ (bias)_._ Dashed lines: lower and upper limits of agreement. Mean difference < 0%: SpO_2_ underestimates SaO_2_. Mean difference > 0%: SpO_2_ overestimates SaO_2_. *SpO*_*2*_ Peripheral oxygen saturation, *SaO*_*2*_ Arterial oxygen saturation

The agreements between measurement of SpO_2_ and SaO_2_ are shown in Fig. 1 and Table 2. The Finger probe measurements underestimated the arterial saturation (SpO_2(pre-exercise)_ = − 0.7% and SpO_2(post-exercise)_ = − 3.3%). At post-exercise, the A_RMS_ was 5.8% and we observed wide limits of agreement between measurement of SpO_2_ and SaO_2_ (95% limit of agreement: − 6–12%).

**Table 2:** Bias, precision and accuracy of peripheral oxygen measurements during 6MWT

Probe location	Number of valid measurements	Bias (SpO_2_–SaO_2_)	Standard deviation	Accuracy (A_RMS_)
Finger				
Pre-test	73	− 0.7%	1.9%	1.9%
Post-test	78	− 3.3%	4.8%	5.8%
Earlobe				
Pre-test	75	1.5%	0.8%	1.7%
Post-test	77	1.3%	2.0%	2.4%
Forehead				
Pre-test	64	0.0%	1.3%	1.3%
Post-test	76	0.2%	1.6%	1.6%

*6MWT* 6-Minute Walk Test, *A*_*RMS*_ Accuracy root mean square

The earlobe and forehead both overestimated the arterial oxygen saturation (earlobe SpO_2(post-exercise)_ = 1.3 and forehead SpO_2(post-exercise)_ = 0.2%). At post-exercise, the A_RMS_ was < 3% and the limit of agreement between measurement of SpO_2_ and SaO_2_ was narrower for the earlobe and forehead than that was seen for the finger probe (earlobe: 95% limits of agreement: -2.5% to 5.2% and forehead: 95% limits of agreement: -2.9% to 3.2%).

### Measurement error of peripheral oxygen measurement

Measurement errors (> ± 4% difference between SpO_2_ and SaO_2_ values) of peripheral oxygen saturation are highlighted with a hollow circle in Fig. 1. At post-exercise, measurement errors were registered in 23%, 3%, and 1% of the patients by the finger, earlobe, and forehead probe, respectively.

In patients with measurement error by the finger probe, 16/20 of patients (89%) had Raynaud’s attack during the 6MWT (*p* = 0.001), and 17/20 of patient (95%) received vasodilator treatment for SSc-related microvasculopathy (*p* < 0.05) (Table 3).

**Table 3:** Clinical and test data of patients with and without measurement error using finger probe at post-exercise (*n* = 78) (Measurement error ± 4% between SpO_2_ and SaO_2_)

	Patients without measurement error using finger probe (*n* = 58)		Patient with measurement error using finger probe (*n* = 20)		*p* value
Variable					
Demographic					
Age, median (IQI)	58	(50–65)	58.5	(52.5–68)	0.60
Sex (female gender), *n* (%)	43	(74.1)	15	(75.0)	1.00
Current smoking, *n* (%)	7	(12.1)	1	(5.0)	0.67
Comorbidity					
Cardiovascular disease*, *n* (%)	4	(6.9)	3	(15.0)	0.37
Diabetes mellitus, *n* (%)	1	(1.8)	2	(10.0)	0.16
COPD and/or asthma, *n* (%)	6	(10.3)	2	(10.0)	1.00
Skin involvement					
Disease duration*, years, median (IQI)	8	(4–14)	8	(4–14)	0.75
Modified Rodnan skin score on digits, mean (SD)	0.9	(0.6)	1.2	(0.7)	0.20
Skin involvement, *n* (%)					
Limited	30	(52)	9	(45)	0.71
Diffuse	27	(47)	11	(55)	
Sine	1	(1.7)	0	(0.0)	
Microvascular involvement					
Raynaud’s Condition Score***, median (IQI)	3	(1–5)	4.5	(2.5- 6)	0.19
History of digital ulcers, *n* (%)^a^	16	(30.2%)	9	(45%)	0.28
Current use of vasodilators, *n* (%)	41	(71%)	19	(95%)	< 0.05*
General capillary density****, mean (SD)	6.9	(1.6)	6.4	(0.9)	0.31
Modified Rodnan skin score on digits, mean (SD)	0.9	(0.6)	1.2	(0.7)	0.20
Raynaud’s phenomenon observed during 6MWT, *n* (%)	25	(43.1%)	18	(90.0%)	< 0.001*
Pulmonary involvement					
Signs of interstitial lung disease on HRCT, *n* (%)	23	(39.7)	12	(60.0)	0.13
Pulmonary arterial hypertension	2	(3.5)	0	(0.0)	0.78

*Cardiovascular disease: ischaemic heart disease, heart failure, atrial fibrillation, stroke and peripheral vascular disease,

**Disease duration among 67/78 who could recall years of first SSc-related symptom (not Raynaud Syndrome), ***73 number of valid measurements,**** 41 number of valid measurements

*6MWT* 6-Minute Walk Test, *IQI* interquartile interval, *SD* standard deviation, *SaO*_*2*_ Arteriel oxygen saturation, *SpO*_*2*_ peripheral oxygen saturation

### Re-test reliability of the minimum peripheral oxygen saturation

**Fig. 2:**
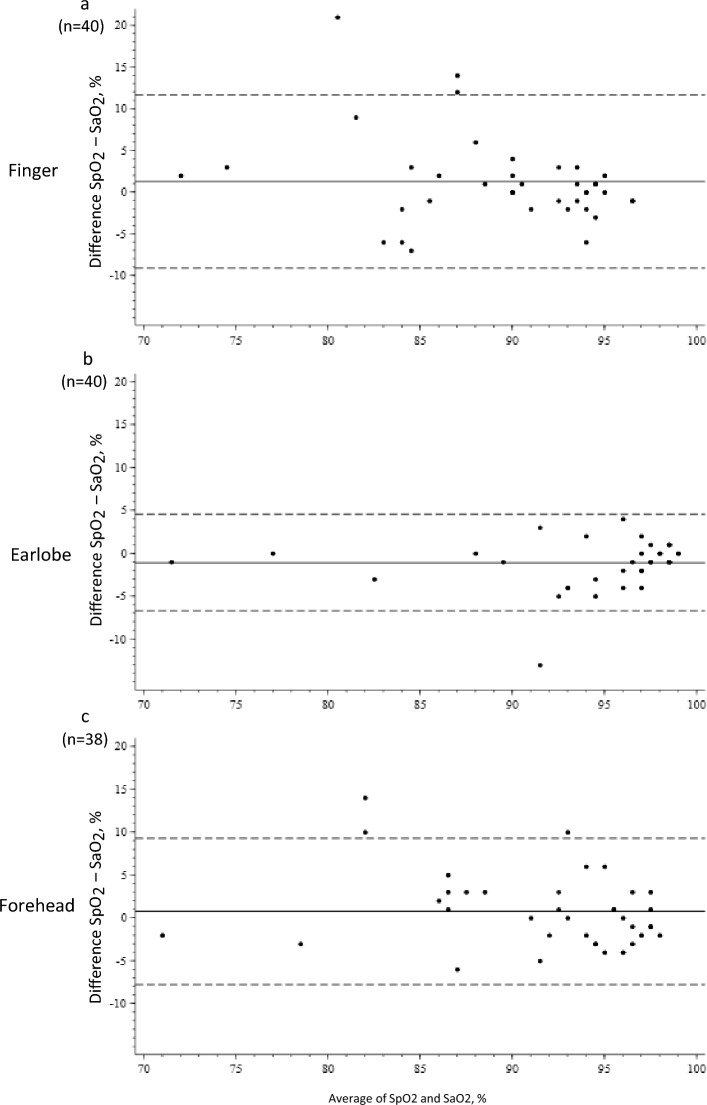
Bland–Altman plots for the re-test reliability of the minimum peripheral oxygen saturation. Solid line: Mean difference between minimum SpO_2_ (visit 2-visit 1). Dashed lines: lower and upper limits of agreement. Mean difference > 0: MinSpO_2_ at visit 2 > minSpO_2_ at visit 1. Mean difference < 0: MinSpO_2_ at visit 2 > minSpO_2_ at visit 1

The mean differences of the minimum SpO2 (visit2-visit1) during the 6MWT was 1% (SD: ± 5), 1% (SD: ± 4) and − 1% (SD: ± 3) for the finger, forehead, and earlobe, respectively (Fig. 2). The ICC showed good agreement using the ear and forehead probe (ICCear = 0.89 [0.80; 0.94]; ICCforehead = 0.77 [0.60; 0.87]), while a modest reliability was found using the finger probe (ICCfinger = 0.65 [0.43; 0.80]). The Mean difference of the 6MWD (visit2-visit1) was 9m (SD: ± 2).

The mean difference of the post-exercise Borg dyspnoea score (visit2-visit1) was 0 (SD: ± 2) (Supplementary Fig. 3).

## Discussion

This study showed that measurement of SpO_2_ using the finger sensor was inaccurate and underestimated the SaO_2_. Furthermore, we demonstrated that SpO_2_ measured at the earlobe and forehead had a high validity and re-test reliability. Indeed, using the finger measurement during the 6MWT resulted in measurement error of saturation in 23% of patients with SSc, with SpO_2_ values being ± 4% different from the SaO_2_.

The poor accuracy of finger measurements of SpO_2_ may be explained by Raynaud’s phenomenon and the peripheral microvasculopathy in patients with SSc, which lead to inaccurate measurements of SaO_2_ due to poor perfusion and hypothermia of the fingers. Indeed, measurement error by the finger probe was primary seen in patients with Raynaud’s attack during the 6MWT and was associated with the use of vasodilator treatment. Several studies have shown impaired perfusion and reduced oxygen delivery in the digital arteries in patients with SSc compared to healthy controls [26–29]. Furthermore, the accuracy of using finger probes in SSc is challenged by variability in measurements of SpO_2_ among fingers in patients with SSc. On the other hand, a recent study reported that blood perfusion of the skin in the face was not different at rest in patients with SSc compared to healthy individuals﻿ [14]. Thus, our findings support that oximetry areas that are not affected by Raynaud’s phenomenon, should be used during the 6MWT in patients with SSc.

*Swigris *et al*.* examined the accuracy of finger SpO_2_ measurement and the prognostic value of desaturation in 83 SSc patients during a cardiopulmonary exercise test [11]. While this study found that the finger SpO_2_ overestimated the SaO_2_, the limits of agreement for the mean difference of finger SpO_2_ and SaO_2_ were wide at maximum exercise as in our study. Furthermore, the study also found that desaturation defined as SpO_2_ below 89% or SpO_2_ fall > 4 points during maximal exercise was associated with a higher mortality. Therefore, it is a crucial finding in our study that the measurement error of the finger probe in a significant proportion of patients had SpO_2_ values being ± 4% different from SaO_2_.

Only one previous study has examined the re-test reliability of peripheral oxygen saturation at different anatomical sites during the 6MWT in patients with SSc. Compared to our study, there was only moderate agreement of forehead and finger SpO_2_ measurements during two 6MWT while the agreement for the earlobe SpO_2_ was poor [7]. Still, this study was small (*N* = 25), and it was only possible to obtain reliable measurements using the earlobe probe in a minority of patients (*n* = 7). In our study, the post-exercise Borg dyspnoea score and the 6MWD were similar at both visits which is in line with other studies that also found good reproducibility of the 6MWD in patients with SSc [7, 30, 31].

The main strength of this study was the large number of prospectively recruited SSc patients and the fact that we were able to compare measurements of continuous SpO_2_ at three different anatomical locations with the arterial saturation as the gold standard. Still, due to the study setup, we were only able to measure arterial oxygen saturation pre- and post-exercise (averagely 20 s post-exercise) and, therefore, the lowest arterial oxygenation may have occurred during the 6MWT. In addition, we were not able to measure oxygen saturation in all patients due to either technical error in collection of data from pulse oximeters or failure in performing or analyzing the blood gas analysis. Last, due to the dynamic performance of the 6MWT, we may not have visually registered all cases of Raynauds’ attacks.

In our study, 43% of the patients had signs of ILD which is similar to the proportion of SSc patients with pulmonary fibrosis in the EUSTAR database [32]. Furthermore, the post-exercise Borg dyspnoea scores were similar to other studies of the 6MWT in patients with SSc [7, 30, 31, 33, 34]. Still, our results are based on only a single center using a specific pulse oximeter. Hence, the disease characteristics of the SSc may be different in other setting and our results may not be representative for other pulse oximetry sensors.

## Conclusion

The present study showed a high accuracy for measuring the SpO_2_ using the earlobe or forehead during the 6MWT in patients with SSc. Furthermore, we demonstrated that finger measurement of SpO_2_ has poor validity and that measurement error of SpO_2_ was associated with markers of peripheral vasculopathy in SSc. The preferred method for monitoring SpO_2_ in clinical SSc-trials and in the monitoring of SSc patients should be at the forehead or the earlobe.

### Supplementary Information

Below is the link to the electronic supplementary material.

Supplementary file1 (DOCX 1218 KB)
