# Supplementary material for: Validity and reliability of measurement of peripheral oxygen saturation during the 6-Minute Walk Test in patients with systemic sclerosis
Source: Rheumatol Int. 2024 Feb 10;44(4):611–20. doi: 10.1007/s00296-024-05532-5 (PMC10914912; doi:10.1007/s00296-024-05532-5)
Supplement: Supplementary file 1 — Supplementary file1 (DOCX 1218 KB) [file 296_2024_5532_MOESM1_ESM.docx]

**SUPPLEMENTARY INFORMATION**

**Article title: Validity and reliability of measurement of peripheral oxygen saturation during the 6-Minute Walk Test in patients with systemic sclerosis.**

*
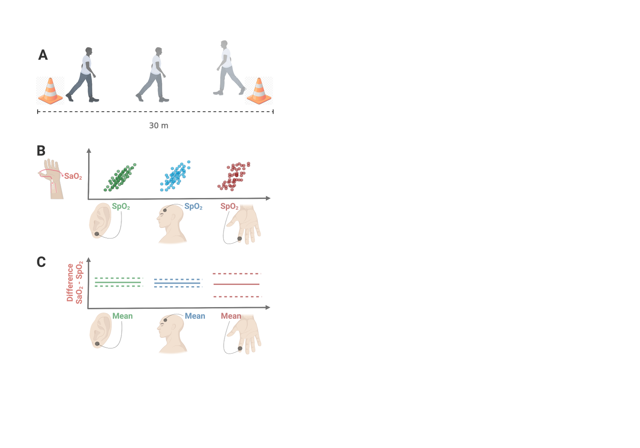
*

**Supplementary Fig 1**: The study setup.

A) During the 6 minute-walk test, patients walked in a 30 meters corridor and turned at every cone. Patients were not allowed to run but allowed to stop to take a break during the test.

B) The 3 pulse oximeters were placed on a finger, forehead, and earlobe on the same patient.

C) The arterial line was placed via ultrasound on the opposite arm of the finger sensor.

Blood draw 1 Blood draw 2


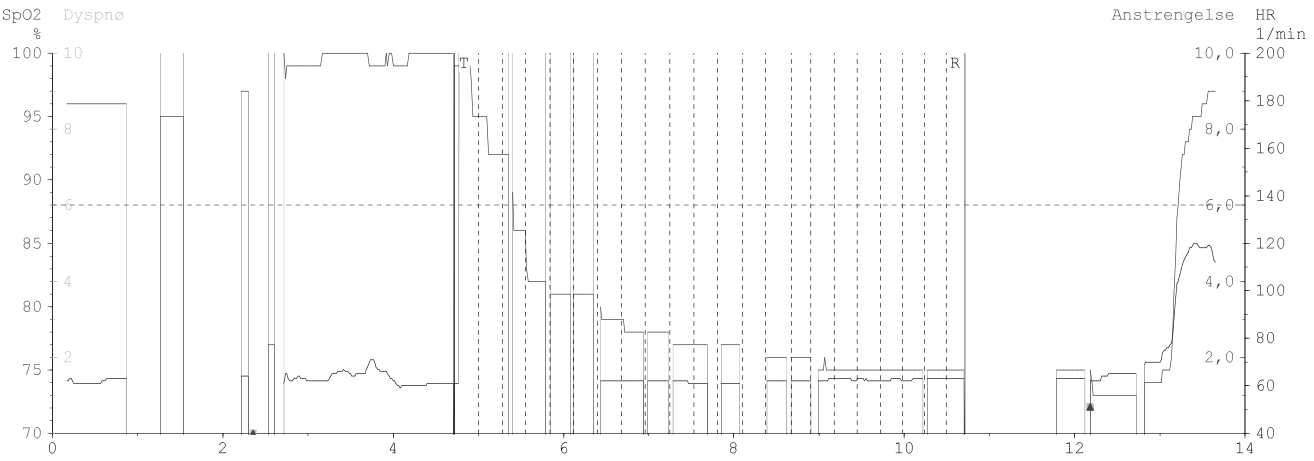

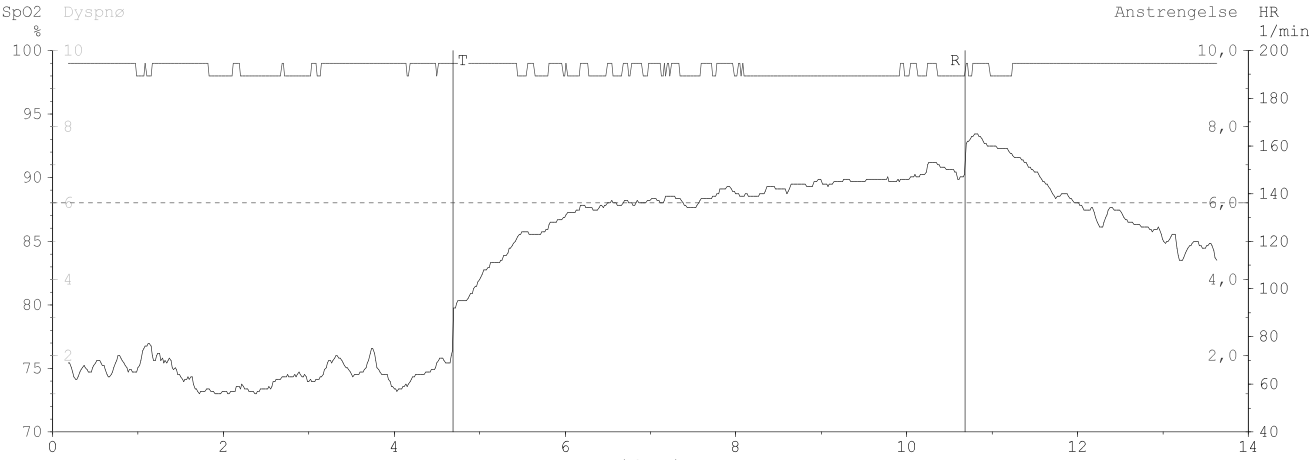

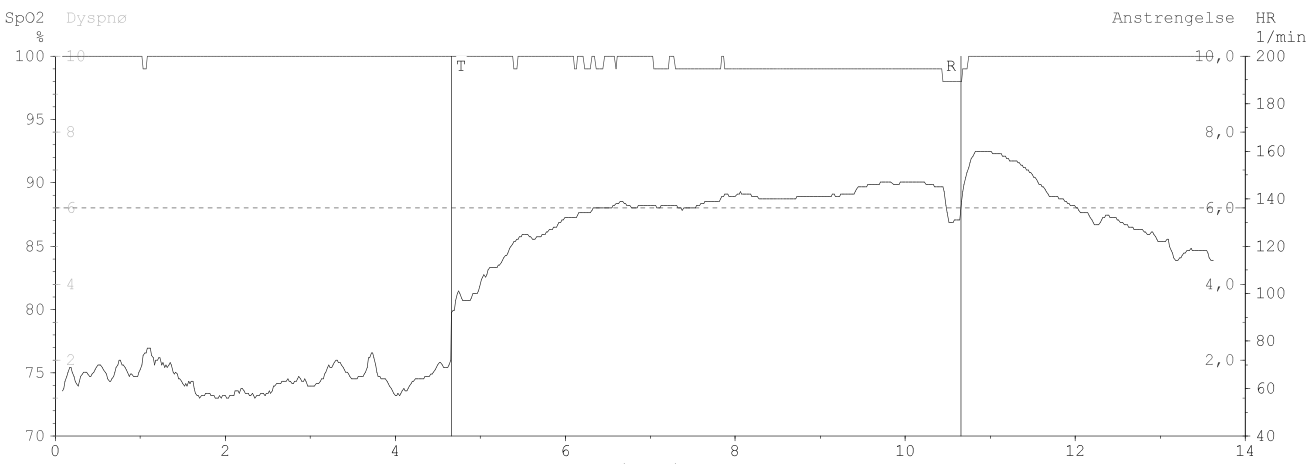


**Finger graph**

SpO2

HR

*Time (min)*

**Forehead graph**

SpO2

HR

*Time (min)*

**Ear graph**

SpO2

HR

*Time (min)*

Pre-exercise The 6MWT Post-exercise

**Supplementary Fig 2**: Graphs with continuous measure of SpO_2_ and HR from a patient with Raynaud’s attack during the 6MWT.

Patient with SSc without known lung disease and no history of smoking.

The SpO_2_ curve of the finger fell steeply during the 6MWT. No corresponding SpO_2_ fall was seen on the earlobe or the forehead. The SaO_2_ was 97% at the time of the second arterial blood gas, while the SpO_2_ was 75%, 98% and 99% for the finger, forehead, and earlobe, respectively. Furthermore, the heart rate (HR) of the finger was much lower during testing (maximal 65 per min) compared to the forehead (maximal 150 per min) and ear (maximal 150 per min). The vertical dashed lines represent the time of the first and second blood draws from the arterial line.Dyspnø (Danish) = Dyspnea (English), Anstrengelse (Danish) = Effort (English).

*Abbreviations:* 6MWT, 6-Minute Walk Test; SpO_2_, Peripheral oxygen saturation; HR, Heart rate.

**Supplementary table 1**. Total number of invalid measurements at pre- and post-exercise (164 measurements).

| **Reason for missing data** | **Total number of invalid measurements (%)** | |
| --- | --- | --- |
| Technical error in collection or transfer of data from pulse oximeter and tablet | Finger | 13 (7.9%) |
|  | Forehead | 24 (14.6%) |
|  | Earlobe | 12 (7.3%) |
| Technical error in performance or analyse of arterial blood gas test | Arterial line | 3 (1.8%) |

| Assessed for eligibility from  (n = 199) | |  | |
| --- | --- | --- | --- |
|  |  | | |
|  |  | | Excluded (n = 118):   - Recent pneumonia (n = 3). - Unable to understand the participant information or unable to perform the exercise test (n= 13) - Declined to participate or not reached by phone (n = 101) |
|  |  | |  |
|  |  | | |
| **Part one: First visit**  Performed 6MWT with arterial line (n = 82) | |  | |
|  |  | | |
| **Part two: Second visit**  Repeated 6MWT without arterial line (n = 40) | |  | |

**Supplementary Fig 3:** Flowchart patient inclusion. Abbreviations: 6MWT, 6-minute Walk Test.

| Supplementary table 2. 6MWT parameters at first visit (n = 82) | | |
| --- | --- | --- |
| Variable | **Value** | |
| Pre-exercise |  |  |
| SaO_2_, %, median (IQI) | 97 | (97 – 98) |
| SpO_2_*, %, median (IQI) |  |  |
| Finger | 97 | (96 – 98) |
| Forehead | 98 | (96 – 99) |
| Ear | 99 | (98 – 99) |
| Borg dyspnoea score, Range 0-10, median (IQI) | 1 | (0 – 1) |
| Post-exercise |  |  |
| SaO_2_, %, median (IQI) | 97 | (96 – 98) |
| SpO_2_*,%, median (IQI) |  |  |
| Finger | 95 | (91 – 97) |
| Forehead | 98 | (96 – 99) |
| Ear | 99 | (98 – 99) |
| Borg dyspnoea score, Range 0-10, median (IQI) (*n* = 81) | 4 | (2 – 5) |
| Minimum SpO_2_ during testing, %, median (IQI) |  |  |
| Finger | 90 | (86 – 94) |
| Forehead | 94 | (90 – 96) |
| Ear | 98 | (95 – 98) |
| Desaturations during 6MWT, *n* (%) |  |  |
| SpO_2_ < 90 % during 6MWT |  |  |
| Finger (*n* = 81) | 35 | (43%) |
| Forehead (*n* = 78) | 18 | (23%) |
| Ear (*n* = 80) | 12 | (15%) |
| SpO_2_ < 88 % during 6MWT |  |  |
| Finger (*n* = 81) | 28 | (35%) |
| Forehead (*n* = 78) | 17 | (22%) |
| Ear (*n* = 80) | 8 | (10%) |
| SpO_2_ < 85 % during 6MWT |  |  |
| Finger (*n* = 81) | 16 | (20%) |
| Forehead (*n* = 78) | 8 | (10%) |
| Ear (*n* = 80) | 3 | (4%) |
| 6MWD, m, median (IQI) | 564 | (502 – 622) |
| Haemoglobin, mmol/L, median (IQI) |  |  |
| Male (*n* = 20) | 9 | (9 – 10) |
| Female (*n* = 61) | 8 | (8 – 8) |
| *SpO_2_ at the time of the blood draw at pre- and post-exercise at first visit.  *Abbreviations:* IQI, Interquartile interval; 6MWT, 6-Minute Walk Test; SaO_2_, Arterial oxygen saturation; SpO_2_, Peripheral oxygen saturation; MinSpO_2_, Minimal SpO_2_; 6MWD, 6-Minute Walk Distance. | | |

 ****

**Supplementary Fig 3: Bland-Altman plot for the re-test reliability of the 6MWD and the post-exercise Borg dyspnoea score**

Y-axis: Mean difference of outcome (visit 2-visit 1, ±1.96 SD). X-axis: Mean of the outcome (visit 2-visit 1).

Mean difference > 0= Outcome at visit 2 > outcome at visit 1. Mean difference < 0= Outcome at visit 2 < outcome at visit 1.

*Abbreviations:* 6MWD, 6-Minute Walk Distance; SD, standard deviation
